# Lab-scale design of two layers wood cellulose filter media to maximize life span for intake air filtration

**DOI:** 10.1038/s41598-021-82855-4

**Published:** 2021-02-04

**Authors:** Qian Yang, Guodong Zhao, Yinglong Wu, Bianzhi Zhang, Jianquan Hu, Lanfeng Hui, Zhong Liu

**Affiliations:** Tianjin Key Laboratory of Pulp and Paper, Tianjin University of Science and Technology, Tianjin, 300457 China

**Keywords:** Engineering, Carbohydrates

## Abstract

The requirement of continuous and stringent growth on filtration performance, including longer life span, higher overall efficiency, lower initial pressure drop and more cost effective, has still drove filter media manufactures to research and develop. One of the possible way to achieve these challenges, was to utilize a dual-channel head-box with two sets of pulp conveying system, which can produce filter media with bulky and gradient properties. In this study, three kinds of commercial cellulose were chosen to make two layers filtration media, analyzed the effect of fiber blend on physical properties and filtration performance. By fine-tune the slurry ratio of top layer, we made one single layer and two layers composition filter media, the thickness and air permeability of composition media were higher than single layer media. According to ISO 5011, filtration performance test has been done to compare single layer media with composition media, this composition gradient profiles that provided the life span 37.0% improvement to the terminal pressure drop during dust injecting, and the dust hold capacity improved 34.7%, the main contributor of dust hold capacity was decided by top layer, however, the overall efficiency was depended on wire side layer.

## Introduction

With the rapid growth of automobile ownership globally every year, huge amount of filter elements, has already consumed plentiful forest and fossil resources to produce^1^. The air filter element was of essential, applied to purify the input air and prevent airborne dust from being sucked into to wear out and shortened the life span of the engine^2^. From the filter media manufacture point of view, the continuous challenge was to develop higher filtration performance filter media, but competitive price^3^. It has already well established in previous literature that multi-layer technology had unique and advantages to result in gradual and relatively slow increase in pressure drop and enlarge engine life time, especially can improve dust hold capacity obviously. Tucny^4^ studied via numerical simulations that clean without fouling process multilayer filter showed limited impact on filtration performance, however, Kaukopaasi^5^ has already confirmed in pilot scale trial that multi-layer media performed better than one single layer media, by which could reduce cost at same filtration performance level using cellulose instead of specialty fibers. Zhang et al.^6^ facilely fabricated multilayered needle-punched/melt blown composite nonwoven filter with good filtration performance regarding on high efficiency, low pressure drop and long service life, which can be good candidate for the removal of PM 2.5. Ling et al.^7^ prepared multilayered nanofiltration membrane by silk nanofibril and hydroxyapatite for water purification with high flux.

Two kinds of multilayer filtration theory were researched for air intake filtration: depth filtration and surface filtration^8,9^. Nanofiber layers working as dust intercept layer fabricated onto supporting layer can enhance filtration performance, the loading dust gathered on nanofiber layer rather than penetrated into filter media, which was benefit for self-clean filter element^10,11^. Long et al.^12^ prepared submicro-fiber composition medium by dual-layers machine, found that after field test, pressure drop of wet-laid submicro-filter was about 45% lower than commercial filter medium and its filtration mechanism was surface fiber capture particles rather than pores. Depth filter media usually composed more than two layers and the potential biggest advance was dust hold capacity improvement^6^. By fabricating of two and three layers of filter media by melt blown and thermal bond fibers, Hasolli^13^ evaluated that top layer made of large fiber size and open fibrous structure, contributed to collect large size particles, and final layer was made of small diameter fibers and low porosity, contributed to remove fine particles. Electro-spinning and micro glass fiber also can be used to prepare composite filter media to enhance filtration efficiency^14,15^. However, these non-renewable and high cost fossil materials were used to fit these advanced methods can be damaged during pleating process, so these methods made air filter element more complicated and expensive for automobile filtration market^16^.

**Figure 1:**
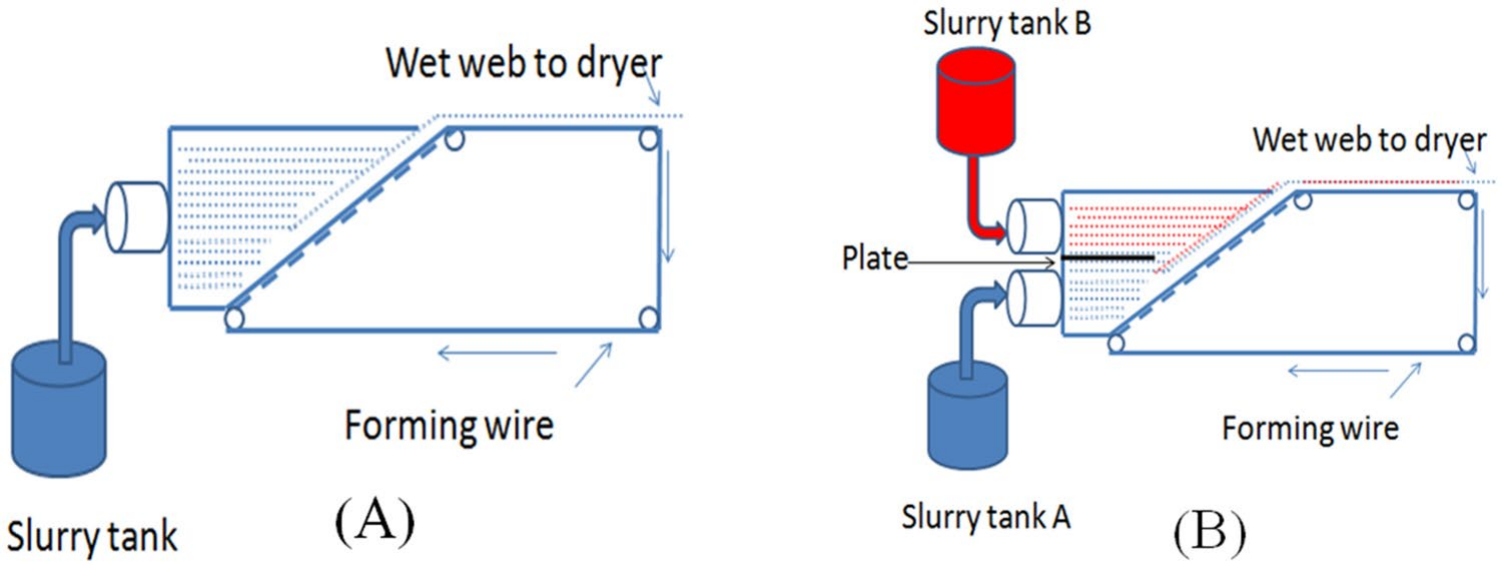
Schematic diagram of single (**A**) and dual-channel (**B**) conveying system head-box.

To the best of my knowledge, there was no article using all commercial natural wood cellulose to design two layers filtration media. To well meet the demand of the filter element market, 100% commercial wood cellulose was used to design and make composition media by lab-scale standard dynamic paper sheet former machine to design two layers air filtration media, which was the most suitable for current practical production. The paper mill only needed to prepare one more pulp conveying system and one plastic plate in head box to separate the slurry of top layer and wire side layer to produce dual-layer composition filtration media, like Fig. 1 illustrated, which has already been commercial producing filter media^17^. The obtained filtration media was tested according to ISO 5011^18^, it was found that the life span to desired pressure drop improved 37%, and dust hold capacity increased 34.7%, the overall efficiency of all the samples were more than 99.5%, which can meet original equipment manufacture’s requirement.

## Results

Three samples, include two composition double layers (Nos. 4 and 5) and one single layer (No. 6) filter media were made with specific fiber blend for each layer to analysis how to effect filtration performance. For the composition media, we designed top layer’s base weight was 30 g/m^2^, and wire side layer’s base weight was 90 g/m^2^. The recipe of top layer was adjusted to make open fibrous structure with wide diameter and long length fiber, which was supposed to intercept large size dust^13^. The fiber blend and the resulted data were listed in Table 1 as followed, for each piece of paper, we made 5 samples, and each sample was tested 3 times to get average data.

**Table 1:** The physical properties of filter media.

Item	1	2	3	4	5	6
Base weight (g/m^2^)	30.45 ± 0.66	30.15 ± 0.73	89.62 ± 1.34	120.01 ± 1.58	120.18 ± 2.08	119.70 ± 3.49
Thickness (μm)	134 ± 16	128 ± 12	388 ± 12	545 ± 18	537 ± 23	514 ± 27
Air permeability (L/m^2^ s)	2100 ± 163	1630 ± 131	508 ± 11	410 ± 28	387 ± 19	350 ± 14
Mean pore size (μm)	N/A^b^	N/A^b^	22.3 ± 0.1	17.8 ± 0.1	17.6 ± 0.3	17.8 ± 0.1

^b^Too big to measure.

Nos. 3 and 6 were one single layer filter media sample, the recipe ratio was pulp 1:pulp 2:pulp 3 = 1:1:1, we can conclude the base weight was positive linearly related with paper thickness, and air permeability was inversely proportional with base weight. During the spraying process, the slurry was deposited onto forming wire round and round smoothly, so the thickness was going higher accordingly.

**Figure 2:**
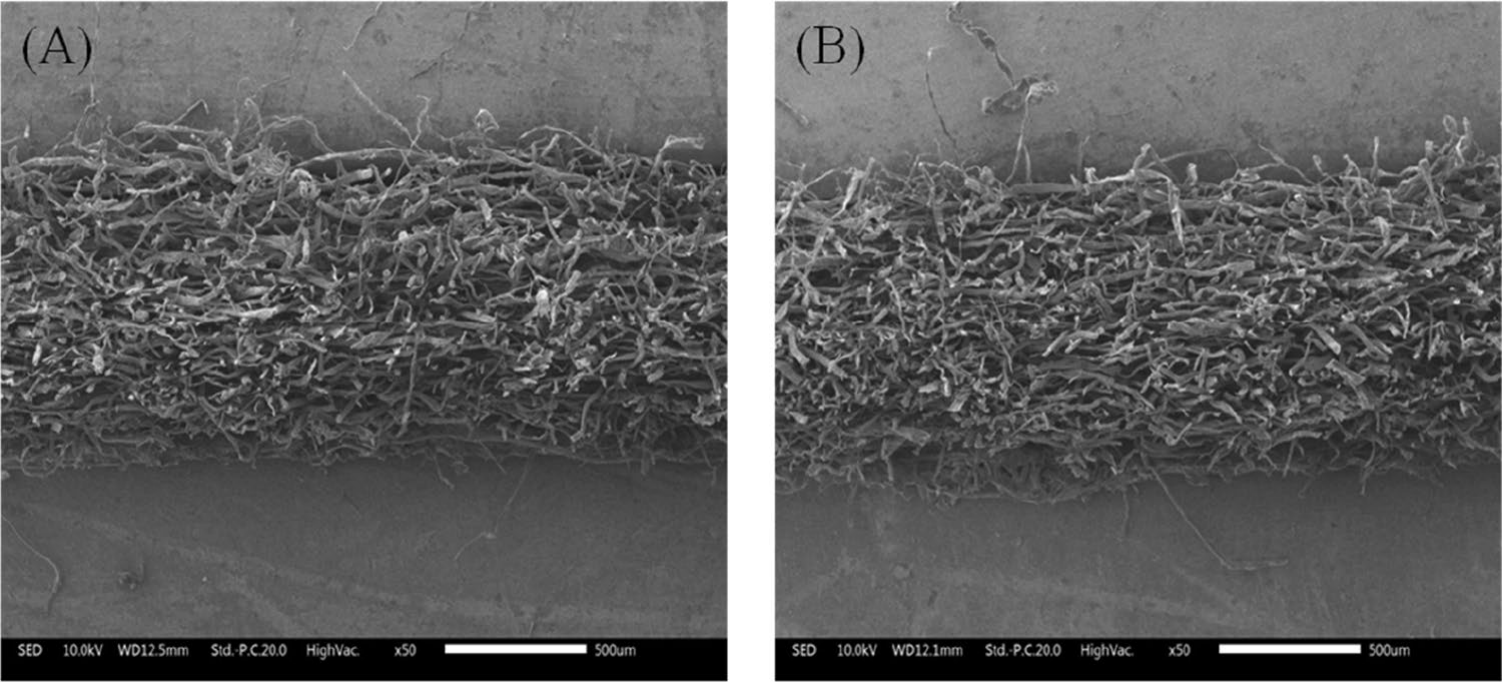
Cross section scanning electron microscopy images of dual layers filter media 4 (**A**) and media 5 (**B**).

Nos. 1 and 2 filter media were controlled as same base weight, however, pulp 1 and pulp 2 were used quantity equally for No. 2 media, and pulp 1 and pulp 3 quantity equally for No. 1 media, therefore, their thickness and air permeability were different because of the air permeability of pulp 1 was higher than pulp 2. From Table 2, we saw that pulp 2 was small diameter and short fiber, pulp 1 was mercerized treated pulp, pulp 3 was course fiber, they were more longer and wider, so we mixed them together to make unique filtration media and analyze their characteristics. We tried to use No. 1 and No. 2 media working as top layer by their open structure to catch course dust, No. 3 media acting as wire side layer, or supporting layer to catch fine size dust. By this design, we got No. 4 and No. 5 sample, it was interesting to note that, even though as same base weight level, the air permeability of No. 4 was higher than No. 5 and the mean pore size kept same level. This gradient structure design, as shown in Fig. 2, can help enlarge filtration performance and save cost of raw materials.

**Table 2:** The characteristics of raw materials.

No.	Species	Length (mm)	Diameter (μm)	Air permeability^a^
Pulp 1	Softwood	2.69 ± 0.04	36.0 ± 0.3	710 ± 25
Pulp 2	Hardwood	0.69 ± 0.01	15.0 ± 0.1	260 ± 12
Pulp 3	Softwood	1.94 ± 0.01	20.6 ± 0.1	550 ± 18

^a^Hand sheet with base weight of 100 g/m^2^.

**Figure 3:**
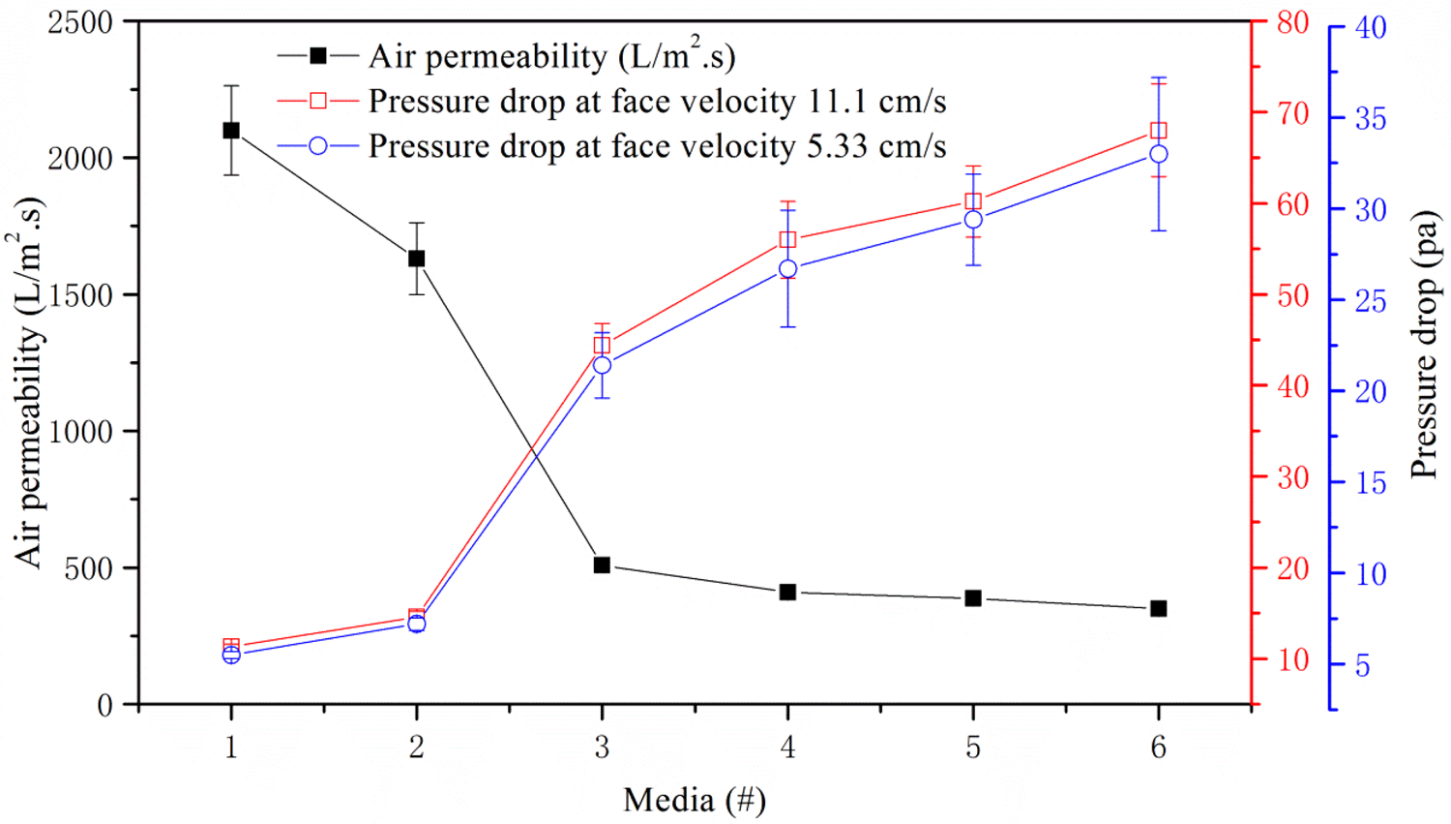
The relationship between pressure drop and air permeability at face velocity 11.1 and 5.33 cm/s.

The initial pressure drop of filter media was tested at face velocity 11.1 and 5.33 cm/s, respectively. Because the currently rated flow of commercial heavy duty truck was 720–1920 m^3^/h normally, and the normal filter element’s extended area was roughly 16 m^2^, so we set up face velocity value 5.33 cm/s for heavy duty truck, also same logic with light duty car to get close with practical application environment. From Fig. 3 when face velocity changed from 5.33 to 11.1 cm/s, at same air permeability level, the pressure drop also became bigger, and the more air permeable, the lower pressure drop when air passed through the paper sheet^19^.

The pressure drop was tested by Palas 3000, according to ISO 5011, the mass concentration of standard SAE fine A2 dust was adjusted as 1000 mg/m^3^, 100cm^2^ sample was placed between two clamps, we can read initial pressure data online at the first 30 s before dust launched.

Pressure drop was decreased with increasing air permeability, the air permeability of Nos. 1 and 2 samples were much higher, we can see the pressure drop data was almost same. The samples of Nos. 3 and 6 were same recipe with different base weight, their pressure drop was inversely proportional with air permeability. No. 6 sample was formed as one layer, which means its top layer and wire side layer’s fiber blend were same, however, Nos. 4 and 5 sample were composition media, the base weight of these two sample were all 30 g/m^2^ but different recipe, No. 4 was more bulky, their wire side were same as No. 3 regarding base weight and recipe, so that the pressure drop were mainly determined by compact layer. Comparison of one layer sample No. 6 and composition samples No. 4 and 5, we can concluded that composition media was benefit to decrease initial pressure drop, which was useful for fuel saving and power strengthening^20^.

**Figure 4:**
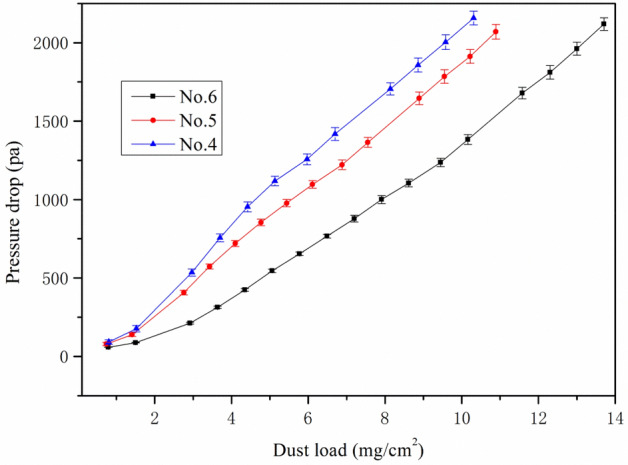
The relationship of dust load with pressure drop at face velocity 11.1 cm/s.

When Palas machine started running to test, the dust generator ejected specific gravimetric dust mass concentration 1000 mg/m^3^ to the untested sample, the initial pressure drop can be read at first 30 s, the termination of the differential pressure was set to add 2000 pa to the initial pressure. The initial pressure was mainly effected by air permeability as talked above, during the process of testing, dust was continuous sprayed onto the filter media, the dust load amount was liner increased, however, we can found from Fig. 4, the No. 6 sample’s pressure drop showed fastest increase at same periods of time, and reached the terminal firstly at 861 s, the composition media No. 4 and No. 5 displayed delay time to reach the terminal, this result can be explained by their media inner structure difference, as we discussed before, the top layer of composition media, Nos. 4 and 5, was more open and bulky showing a fibrous structure, which caused longer dust loading time and showed higher amount of dust load at same base weight level, as details listed in Table 3.

**Table 3:** Summary of tested filtration performance data.

	Unit	No. 4	No. 5	No. 6
Measurement duration	s	1180 ± 6	1016 ± 6	861 ± 6
Dust hold capacity	g/m^2^	132 ± 3	108 ± 1	98 ± 1
Overall efficiency	%	99.57 ± 0.02	99.66 ± 0.01	99.59 ± 0.02

**Figure 5:**
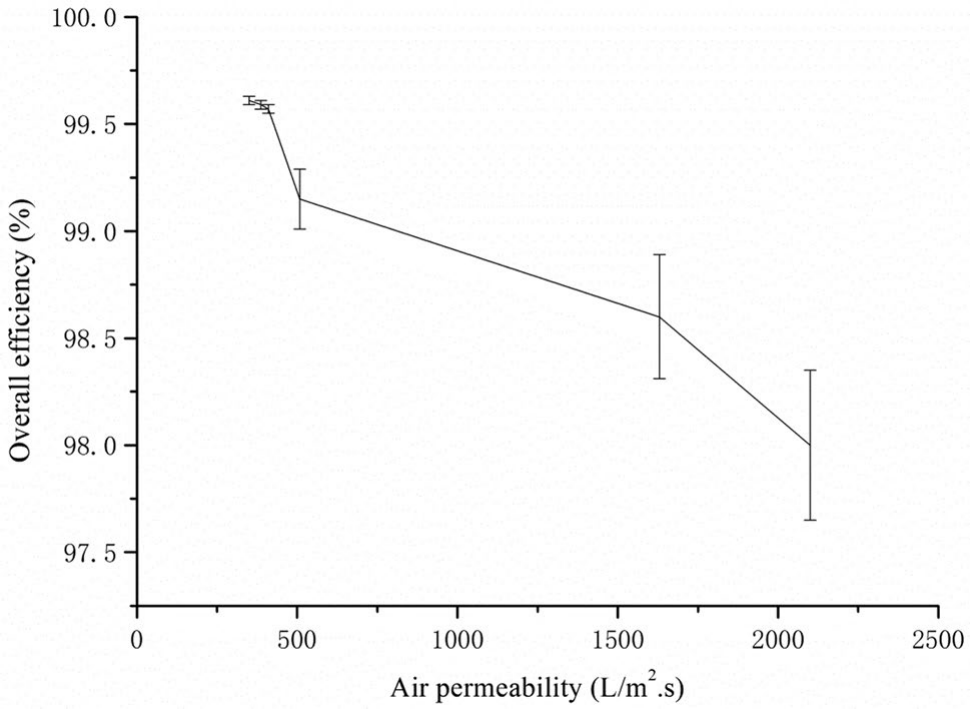
The relationship of overall efficiency and air permeability at face velocity 11.1 cm/s.

The overall efficiency of filter media was determined by wire layer, which was higher density made of small diameter fiber, from Table 1 we knew that the mean pore size was correlated with air permeability, and from Fig. 5, the higher air permeability resulted lower overall efficiency, which was consistent with general result^21^.

**Figure 6:**
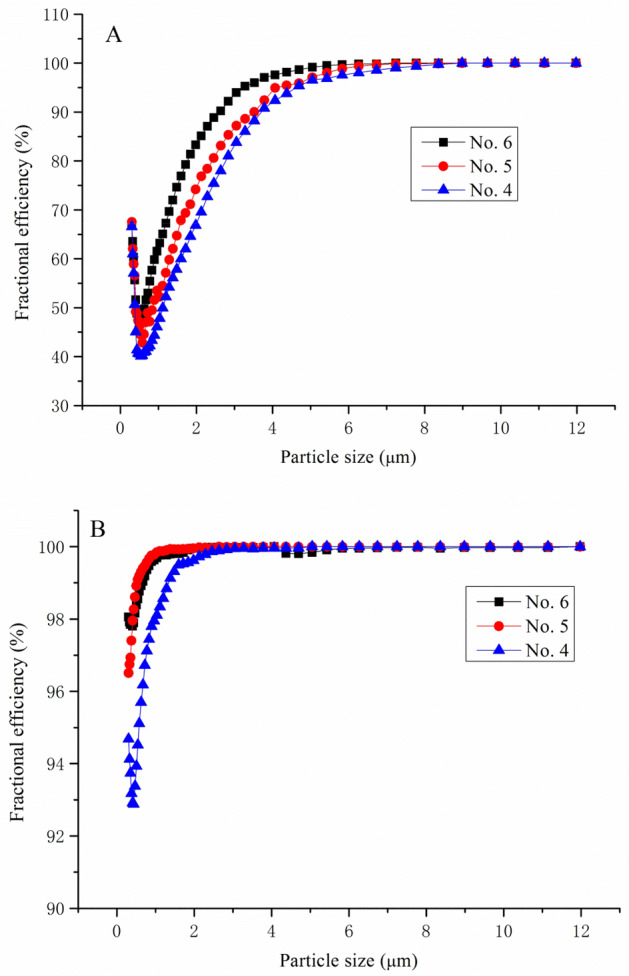
Fractional efficiency of different particle size at 69 s (**A**) and 321 s (**B**) at face velocity 11.1 cm/s.

**Figure 7:**
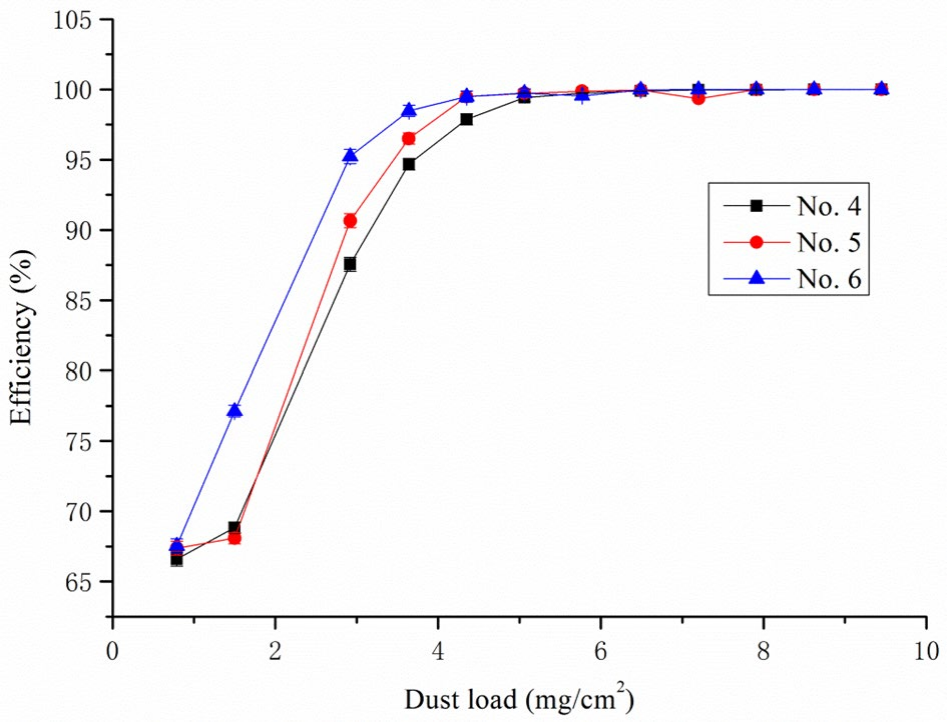
Overall efficiency of 0.305 μm particle size dust at face velocity 11.1 cm/s.

The fractional efficiency of particle size from 0.305 to 12 μm was conducted by two Welas 2070 in-line particle counter, located at upstream and downstream of filter media, respectively, to calculate the number of various size of particle before and after passed through the filter media. As shown in Fig. 6, in the first 69 s, dust was ejected from dust generator onto the clean filter media at the face velocity 11.1 cm/s, the retention reason of fine size particle (< 1 μm), according to filtration theory, was brownian diffusion and interception by fibers itself, 30–60% of fine particle can cross the maze-like pore structure, however, large size dust (> 5 μm) can be captured by interception more than 90% at the first 69 s. After 321 s, the efficiency of fine dust was more than 90%, because according to cake filtration theory the dust layer has already formed, which can act as filtration layer to intercept fine and large size dust, same as the pressure drop increased over time. Also from result of overall efficiency of 0.305 μm particle size, as shown in Fig. 7, we can see from the beginning test before the dust layer formation, about 65% of fine dust can be trapped by cellulose fibers, when the dust was captured, the filter media pressure drop started to rise because the clogged pore size, and the efficiency improved to more than 99.5% after 600 s. The air permeability and thickness of No. 6 media indicated its higher solidity caused by finer fiber, which was beneficial for fine particles retention by internal impaction and interception^22^.

## Discussion

The filtration performance of composition media made by lab scale paper machine showed 4–6% higher thickness and 10–17% higher air permeability at same base weight compared with single layer media, which mostly attributed to the top layer modified by specific fiber blend. The unique characteristic of bulky structure caused 37.0% enhancement of longer life span and excellent filtration performance regarding on dust hold capacity improved 34.7% and overall efficiency was all kept more than 99.5%. The filtration efficiency was determined by mean pore size, and the fine and course dust can pass through filter media mainly happened in the early stage of testing, at the first 69 s, the fine dust can pass through the media, after 321 s, more than 90% of fine dust can be intercepted by interception between the fiber itself and formed layer.

## Methods

### Materials

In order to obtain various properties of air filtration paper, we chose 3 kinds of raw pulps. Their basic physical properties as followed in Table 2, the length and diameter of pulps were tested by Fiber Quality Analyzer (Fiber Tester 912, L&W, Sweden). The standard A2 size fine dust (ISO 12103-1) was used for filtration performance test by Palas MFP 3000 (PALAS, Germany) followed by the ISO 5011.

### Characterization

The thickness of hand sheet was measured according to TAPPI T411, the air permeability was tested according to ASTM D737 under the pressure 200 Pa, the maximum and mean pore size was tested by capillary pore diameter measuring instrument (Porolux 100, Porometer NV, Belgium) according to ASTM F316, the surface tension of infiltrating fluid was 16 mN/m. The filtration performance was tested by PALAS MFP 3000, according to ISO 5011 at the face velocity was 5.33 and 11.1 cm/s, respectively. The terminal pressure drop was 2000 pa. The dust hold capacity was gravimetric weighted before and after dust loaded, the filtration efficiency was conducted by two particle counter, and initial pressure can be read online at first 30 s from dust depositing. The morphology of resulted hand sheet was observed by scanning electron microscope (JSM-IT300LV, Hitachi, Japan).

### Preparation of filter paper

Standard dynamic paper sheet former machine (BP251, TECHPAP SAS, France) was used to simulate the two sets of slurry conveying systems in head-box of the paper machine. The specific fiber blend of wire side layer was prepared and sprayed onto forming wire, and then top side layer slurry was sprayed and deposited on top layer. The each layer and total base weight was designed and controlled by slurry amount, after wet paper web formed, the filter paper was vacuum dried and stored at constant temperature and humidity room for test.
